# Field Screen and Genotyping of *Phaseolus vulgaris* against Two Begomoviruses in Georgia, USA

**DOI:** 10.3390/insects12010049

**Published:** 2021-01-10

**Authors:** Gaurav Agarwal, Saritha Raman Kavalappara, Saurabh Gautam, Andre da Silva, Alvin Simmons, Rajagopalbabu Srinivasan, Bhabesh Dutta

**Affiliations:** 1 Department of Plant Pathology, Coastal Plain Experimental Station, University of Georgia, Tifton, GA 31793, USA; Gaurav.Agarwal@uga.edu (G.A.); SarithaRama.Kavalappara@uga.edu (S.R.K.); 2 Department of Entomology, University of Georgia, 1109 Experiment Station, Griffin, GA 30223, USA; sg37721@uga.edu (S.G.); babusri@uga.edu (R.S.); 3 Department of Horticulture, Coastal Plain Experimental Station, University of Georgia, Tifton, GA 31793, USA; adasilva@uga.edu; 4 U.S. Vegetable Laboratory, Agricultural Research Service, United States Department of Agriculture, Charleston, SC 29414, USA; Alvin.Simmons@usda.gov

**Keywords:** cucurbit leaf crumple virus, sida golden mosaic Florida virus, whitefly, snap beans, lima beans

## Abstract

**Simple Summary:**

Snap bean (*Phaseolus vulgaris*) production and quality have been negatively impacted by two whitefly-transmitted begomoviruses: cucurbit leaf crumple virus (CuLCrV) and sida golden mosaic Florida virus (SiGMFV), which often appear as a mixed infection in Georgia. However, there is no information available in terms of resistance to these two viruses in commercial cultivars/genotypes. Hence, commercially available snap bean varieties/genotypes (*n* = 84 in 2018; *n* = 80 in 2019; most of the genotypes were common in both years (with a few exceptions) were screened in two field seasons of 2018 and 2019. We also included two commonly grown Lima bean (*Phaseolus lunatus*) varieties in our field screening. As a result of this screening, we identified twenty *Phaseolu*s genotypes with high-to-moderate levels of resistance and twenty-one genotypes with high levels of susceptibility. While there were differences among the *Phaseolus* spp. in severity of viral symptoms, suggesting differential susceptibility to viruses (CuLCrV and SiGMFV) and potential field resistance, the resistance mechanism is yet to be characterized. However, based on the greenhouse evaluation with two genotypes-each (susceptible vs. resistant) exposed to viruliferous whiteflies infected with CuLCrV and SiGMFV, we observed that the susceptible genotypes accumulated higher copy numbers of both viruses and displayed severe crumple severity compared to the resistant genotypes, indicating that resistant might potentially be against the virus complex than against the whiteflies. Adult whitefly counts differed among the *Phaseolus* spp. in both the years, indicating variability in host preference. We further sequenced 82 genotypes (80 snap bean and two Lima bean) to unravel the variations within the genomes. Genome sequencing followed by bioinformatic analyses revealed a considerable number of sequence variants, single nucleotide polymorphisms (SNPs), and insertions and deletions (InDels) in the genomes. Considering the variations in disease response and the underlying variations in the sequenced genomes, it can be speculated that some of the phenotypic variations (against CuLCrV and SiGMFV) could be due to a high level of genomic variation in the host. Future genome-wide association studies with the identified genomic variants may shed some light on this.

**Abstract:**

The production and quality of *Phaseolus* *vulgaris* (snap bean) have been negatively impacted by leaf crumple disease caused by two whitefly-transmitted begomoviruses: cucurbit leaf crumple virus (CuLCrV) and sida golden mosaic Florida virus (SiGMFV), which often appear as a mixed infection in Georgia. Host resistance is the most economical management strategy against whitefly-transmitted viruses. Currently, information is not available with respect to resistance to these two viruses in commercial cultivars. In two field seasons (2018 and 2019), we screened *Phaseolus* spp. genotypes (*n* = 84 in 2018; *n* = 80 in 2019; most of the genotypes were common in both years with a few exceptions) for resistance against CuLCrV and/or SiGMFV. We also included two commonly grown Lima bean (*Phaseolus lunatus*) varieties in our field screening. Twenty *Phaseolus* spp. genotypes with high to moderate-levels of resistance (disease severity ranging from 5%–50%) to CuLCrV and/or SiGMFV were identified. Twenty-one *Phaseolus* spp. genotypes were found to be highly susceptible with a disease severity of ≥66%. Furthermore, based on the greenhouse evaluation with two genotypes-each (two susceptible and two resistant; identified in field screen) exposed to viruliferous whiteflies infected with CuLCrV and SiGMFV, we observed that the susceptible genotypes accumulated higher copy numbers of both viruses and displayed severe crumple severity compared to the resistant genotypes, indicating that resistance might potentially be against the virus complex rather than against the whiteflies. Adult whitefly counts differed significantly among Phaseolus genotypes in both years. The whole genome of these *Phaseolus* spp. [snap bean (*n* = 82); Lima bean (*n* = 2)] genotypes was sequenced and genetic variability among them was identified. Over 900 giga-base (Gb) of filtered data were generated and >88% of the resulting data were mapped to the reference genome, and SNP and Indel variants in *Phaseolus* spp. genotypes were obtained. A total of 645,729 SNPs and 68,713 Indels, including 30,169 insertions and 38,543 deletions, were identified, which were distributed in 11 chromosomes with chromosome 02 harboring the maximum number of variants. This phenotypic and genotypic information will be helpful in genome-wide association studies that will aid in identifying the genetic basis of resistance to these begomoviruses in *Phaseolus* spp.

## 1. Introduction

Among commonly grown *Phaseolus* spp., *Phaseolus vulgaris* L. (common bean, snap bean) is an annual legume crop with a diploid genome size of 521.1 Mb (*2n* = 22) [1]. Snap bean is one of the most important affordable food legumes for humans [2], which is consumed by over 80 million poor people in regions of Latin America, the Caribbean, and Eastern and Southern Africa. In the U.S., snap bean is an important horticultural crop, especially for the state of Georgia where snap bean is grown in 9979 acres and generates an annual revenue of $24 million dollars [3]. However, the production and quality of snap bean have been negatively impacted by two whitefly-transmitted begomoviruses, namely cucurbit leaf crumple virus (CuLCrV) and sida golden mosaic Florida virus (SiGMFV), which often appear as a mixed infection in Georgia [4].

CuLCrV is a bipartite begomovirus first identified in watermelon in the Imperial Valley of southern California in 1998 [5] and in Georgia in snap beans in 2009 [6]. In August 2018, snap beans with characteristic begomovirus infection symptoms (crumpled, curled, and thickened leaves) were found in Tifton, Georgia, and these plants were heavily infested with whiteflies. Subsequent analysis with degenerate and specific begomovirus primers revealed the presence of SiGMFV in infected plant tissues. In the southeastern US, SiGMFV (a bipartite virus) was first reported in Florida in 2006 on snap beans with infected plants displaying leaf mottling, puckering, and severe curling symptoms [7]. Both the viruses are transmitted by the sweet-potato whitefly (*Bemisia tabaci* Gennadius), which is a predominant vector of begomoviruses in the farmscapes of Georgia [8]. Currently, leaf crumple disease management is centered on vector control, which usually occurs via insecticides. Disease management via vector control is unreliable and insufficient. On the contrary, host-resistance is the more economical and sustainable approach that can potentially minimize field infestation, but there is a considerable lack of information on host resistance against these two begomoviruses on snap bean in the US. Moreover, among the commercially available *Phaseolus* spp., including snap bean and Lima bean, there is a lack of background information on the level of field resistance against these begomoviruses that are prevalent in the southeastern US particularly in Georgia.

Comprehensive understanding of the genetics of host resistance is necessary for breeding resistant varieties, which involves identifying markers and genes that confer resistance. Single nucleotide polymorphisms (SNPs) have high a frequency of occurrence throughout the genome and are considered as preferable genetic markers in breeding for disease resistance. SNPs, along with longer sequence variants, are insertions and deletions (InDels), aided in the discovery of quantitative trait loci (QTLs) and genes associated with disease resistance and agronomic traits in many cultivated crops. Prior genomic studies on *P. vulgaris* (dry beans) focused on agronomic and abiotic stress related traits (drought stress and salt tress), but none of them focused on identifying resistance to viral pathogens. Biparental QTL mapping and genome wide association studies (GWAS) have been used to discover such traits in common bean [9,10,11]. The information on genetic variation in the commercially available *Phaseolus* spp. cultivars is lacking, which can be utilized in GWAS and may potentially aid in identifying the genetic basis of resistance. The objectives of this manuscript are to evaluate whether commercially available *Phaseolus* spp. possess resistance to leaf crumple disease (caused by begomovirus complex; CuLCrV and SiGMFV) under field conditions, characterize resistance under greenhouse conditions and assess and identify genetic variations among them or not. In the current study, besides evaluating the response of *Phaseolus* spp. to natural infection of begomoviruses under field conditions for two consecutive years (seasons), sequence variants (SNPs and Indels), and their distribution in the *Phaseolus* cultivars, were also identified using whole-genome sequencing (WGS), which will form the foundation for future studies in identifying the genetic basis of resistance to CuLCrV and SiGMFV.

## 2. Materials and Methods

### 2.1. Plant Materials

Eighty-four *Phaseolus* genotypes, including 82 snap bean and two Lima bean (*P. lunatus*) genotypes, were used in 2018. Two Lima bean genotypes that are close relatives of snap beans, Jackson wonder and Fordhook, were also included. Eighty genotypes were tested in 2019, of which seventy-six genotypes were the same as those tested in 2018. Seeds of BMN- RMR- 13, Bronco 2, Lakatte, SB4734, SB4735, SB4744, SB4679, and SV1137 were not available for evaluation in 2019. Hence, four genotypes of snap beans, Achiever, Blue Lake 274, Coyote and Greenback, were only evaluated in 2019 (Table 1). Seeds were collected from commercial seed companies and Germplasm Resource Information Network (GRIN) of the United States Department of Agriculture (USDA) (Table 1).

### 2.2. Experimental Design, Layout and Environmental Conditions

The genotypes mentioned above were evaluated for resistance to CuLCrV and SiGMFV under field conditions at the University of Georgia, Tifton. In both years (2018 and 2019), seeds were grown in 12 individual 138 m long-raised beds. Each raised bed was divided into plots with dimensions of 3.04 m × 0.91 m. Each plot was comprised of 20 plants planted in an in-row spacing of 7.62 cm, double rows spaced at 46 cm were used within each bed. Treatments (genotypes) were replicated (r = 3) using a randomized complete block design. Natural whitefly infestation relied upon virus transmission and resultant disease. The whitefly pressure was considerably higher in the 2019 field season compared to 2018. Fields were irrigated with overhead irrigation, twice per week or as needed depending on rainfall. All cultural practices and disease management followed the UGA Cooperative Extension recommendations [12]. Insecticides were not sprayed in order to ensure the survival of whiteflies for disease incidence and spread. Averages of maximum and minimum temperatures in 2018 during the growing period were 34.5 °C and 21.1 °C, respectively, with an accumulated precipitation of 0.25 cm. In the 2019 growing period, averages of maximum and minimum temperatures were 32.5 °C and 22.5 °C and the accumulated precipitation was 0.23 cm.

### 2.3. Response of Phaseolus spp. (Snap Beans and Lima beans) Genotypes to Leaf Crumple Disease in the Field

**Figure 1:**
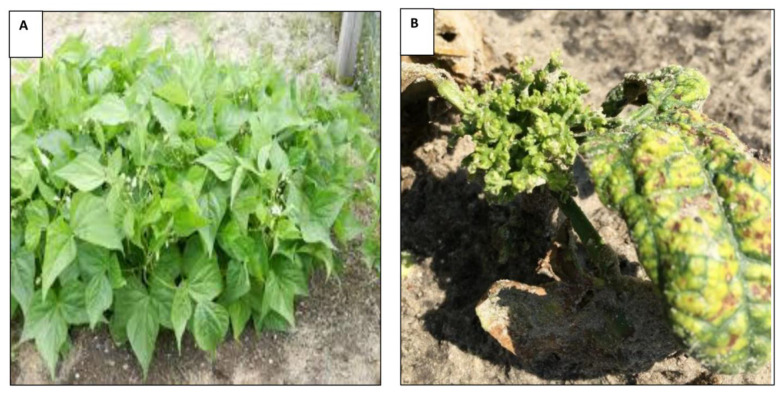
Symptoms of leaf crumple on *P. vulgaris* plants under field conditions. A non-infected *P. vulgaris* (**A**) and an infected plant (**B**) with severe stunting, leaf crumpling and distortion, and leaf mosaic symptoms.

In 2018, evaluation of genotypes for virus resistance was conducted at 30 days after sowing (DAS). Since Hurricane Michael destroyed the crop in early October 2018, a second evaluation of resistance was not possible. In 2019, leaf crumple disease evaluation was conducted twice, at 30 and 45 DAS. For each genotype, plants were evaluated visually for disease incidence and severity. Disease severity in five randomly selected plants per plot per genotype was evaluated using a severity scale of 0 to 100. A plant with no crumpling, mosaic and stunting was scored as 0 (Figure 1A). A plant with severe leaf crumpling, mosaic and stunting was scored as 100 (Figure 1B). Genotypes with disease severity ≤20% were rated as highly resistant, 21%–50% as moderately resistant, 51%–65% as susceptible and ≥65% as highly susceptible.

### 2.4. Whitefly Count

Adult whiteflies were counted at 30 DAS in 2018 and 45 DAS in 2019 on each genotype. Counting was conducted in the field on the lower side of leaves in the morning hours when whiteflies are not very active. Whiteflies adults were enumerated on the top three, fully expanded leaves by gently turning the leaf over by the tip. Whitefly counts were taken from 15 plants for each genotype, five from each replicate. Whitefly count data for 2018 and 2019 were analyzed independently using the linear mixed model in software R version 3.4.2. Genotypes were considered as fixed effects and replicates were considered as random effects. To meet the assumption of ANOVA (normality and homoscedasticity of variance) prior to analysis, data were log(X + 1) transformed. After transformation, assumptions of ANOVA were met at *p* = 0.01. Post-hoc analyses were performed using the “emmeans” package with the default Tukey’s honest significant difference test (*p* = 0.05).

### 2.5. DNA Isolation, Library Preparation, Sequencing and Quality Filtering of Raw Data

A total of 82 *Phaseolus* genotypes (80 snap beans and two Lima beans) were sequenced. Total DNA was isolated from a single plant of each genotype collected arbitrarily from the field using DNEasy plant mini kit (Qiagen, Düsseldorf, Germany) following the manufacturer’s instructions. A 50 ng/µL of DNA per sample was used for library preparation. Genomic DNA of each sample was randomly sheared into short fragments of about 300–500 bp. The obtained fragments were subjected to library construction using the NEBNext^®^ DNA Library Prep Kit (New England BioLabls, Ipswich, MA, USA), strictly following the instructions. After the end repairing, dA-tailing, and further ligation with NEBNext adapter, the required fragments (in 300–500 bp size) were PCR enriched by P5 and indexed P7 oligos. Library was subsequently sequenced on NovaSeq 6000 platform (Illumina, San Diego, CA, USA). The original sequencing data acquired by NovaSeq 6000 recorded in image files were firstly transformed to sequence reads by base calling with the CASAVA software to generate FASTQ files. Pair-end sequencing were performed with the read length of PE150 bp at each end. The raw FASTQ reads obtained were quality filtered. We discarded the paired reads when either read contained adapter contamination, when uncertain nucleotides (N) constitute more than 10 percent of either read, and when low quality nucleotides (base quality less than 5, Q ≤ 5) constitute more than 50 percent of either read.

### 2.6. Mapping of Filtered Read Data on the Reference Genome and Variant Calling

The filtered sequencing data was aligned on *Phaseolus vulgaris* reference genome (Pvulgaris_442_v2.0_softmasked) available at the legume information system (LIS). BWA software [13] (parameters: mem -t 4 -k 32 -M) was used for alignment and the mapping rate and coverage were counted according to the alignment results. The binary alignment (.bam) data files are available at NCBI under BioProject ID PRJNA680977. The duplicates were removed by SAMtools. Individual SNP variations were detected using GATK. SNPs and InDels were further filtered based quality and depth. All variants with Qual < 30, SOR > 3.0, DP < 6, heterozygous and multi-allelic calls were filtered out. Filtered SNPs and InDels were annotated using Annovar [14]. SNP and InDel densities per kb were calculated in 100 kb bins all throughout the 11 chromosomes of *P. vulgaris*.

### 2.7. Confirmation of Begomoviruses (CuLCrV and SiGMFV) Infection Associated with Leaf Crumple Symptoms in Phaseolus spp.

In order to ensure if the symptoms observed were associated with begomoviruses, we tested *Phaseolus* spp. leaf samples from forty randomly collected genotypes that displayed symptoms from the field for two years. Symptomatic leaf samples from three plants per replicate per genotype were tested for the presence of CuLCrV and/or SiGMFV. Total DNA from 100 mg symptomatic leaf tissues was extracted using GeneJET Plant Genomic DNA Purification Kit (ThermoFisher Scientific, Waltham, MA, USA) following the manufacturer’s protocol. Presence of CuLCrV was tested via qPCR using the primers (forward 5′-CCTCAAAGGTTTCCCGCTCT-3′ and reverse 5′-CCGATAGATCCTGGGCTTCC-3′), which amplify a 110 bp region of coat protein gene using protocol and cycling conditions, as mentioned earlier by Gautam [4]. For SiGMFV, primers SiGMFV-QF and SiGMFV-QR [4], which targeted a 114 bp region of DNA-A of SiGMFV, were used. DNA samples tested positive for CulCrV and SiGMFV earlier were included as positive controls. Water was added in place of DNA in negative controls.

### 2.8. Accumulation of CuLCrV and SiGMFV and Leaf Crumple Severity in P. vulgaris Genotypes (Susceptible vs. Resistant; Identified in Field Screen) when Exposed to Viruliferous Whiteflies (Mixed Infected with CuLCrV and SIGMFV) under Greenhouse Conditions

Two highly susceptible (Top crop and Gold mine) and two resistant (Sybaris and Prevail) varieties (based on 2018 field evaluation) were assessed for both SiGMFV and CuLCrV accumulation under greenhouse conditions. Ten symptomatic squash (*Cucurbita pepo*; cv. Goldstar) and ten symptomatic prickly sida (*Sida rhombifolia*) were obtained from a research farm at UGA, Tifton and their infection status for the presence of CuLCrV and SiGMFV were confirmed using specific PCR assays for both viruses, as described earlier. After ascertaining the presence of only CuLCrV in squash and only SiGMFV in prickly sida, plants carrying each viral pathogen were kept in whitefly-proof cages under greenhouse conditions of 25 °C and 60% relative humidity (RH) with a photoperiod of 14 h of light and 10 h of darkness. Adult whiteflies that were maintained on cotton (in a separate greenhouse) were collected and exposed to symptomatic squash and prickly sida plants. Two hundred whiteflies were exposed to each symptomatic host plant. After a 48 h acquisition access period on each host plant (squash or prickly sida), whiteflies were collected and inoculated (*n* = 50 viruliferous whiteflies each with CuLCrV and SiGMFV per Phaseolus genotype) on to tested three-week old Phaseolus genotypes (Top crop, Gold mine, Sybaris and Prevail). Viruliferous whiteflies carrying CuLCrV or SiGMFV from squash or prickly sida, respectively, were clip-caged and an inoculation access period (IAP) of 48 h was provided. All whiteflies were removed post 48 h IAP, and plants were sprayed with Admire Pro (Bayer Crop Science LP, Research Triangle Park, NC, USA) to kill any remaining whiteflies. Six replicates per snap bean line were assessed in two independent experiments. Inoculated snap bean plants were kept in whitefly-proof cages for 30 days and accumulation of CuLCrV and SiGMFV were determined at 30-days post-inoculation (DPI) using a quantitative PCR (qPCR) assay, as described earlier. Phaseolus genotypes were also inoculated with whiteflies (same as above) that were given 48 h acquisition access on non-infected squash or prickly sida plants maintained under the same conditions as described above.

Leaf tissue (50 mg per replicate per genotype) were subjected to genomic DNA extraction using Qiagen Plant Genomic DNA extraction kit (Qigen, Düsseldorf, Germany) and 10 ng of DNA was used as a template for qPCR. Each sample was tested in duplicate, and absolute CuLCrV or SiGMFV were quantified using a standard curve, as described earlier [15]. Separate plasmid copies for CuLCrV or SiGMFV were generated and the number of copies were measured using a formula described by Gadhave et al. [16]. The differences in CuLCrV or SiGMFV accumulation in each genotype were analyzed using a Kruskal-Wallis test in the SAS 9.4 package. Plants were also assessed for leaf crumple symptoms at 30 DPI using a severity scale of 0–100, where 0 = no visible symptoms observed; 20 = no stunting, mild foliar chlorosis, mild internodal shortening, and normal flowering; 40 = mild stunting, foliar chlorosis, moderate internodal shortening, and reduced flowering; 60 = moderate stunting, severe chlorosis, reduced flowering, and poor pod setting; 80 = severe stunting, severe foliar chlorosis, severe internodal shortening, flowering severely affected or no flowering and no pod setting; 100 = plant death. Analysis of variance was conducted to determine the effect of genotypes on disease severity, and Fischer’s Least Significant Difference (LSD) test (*p* < 0.05) was used for mean separations.

## 3. Results

### 3.1. Response of Phaseolus spp. (Snap Beans and Lima Beans) Genotypes to Leaf Crumple Disease in the Field

In both years, typical symptoms of virus infection included yellow mosaic, leaf crumpling, and shortening in varying degrees in different genotypes (Figure 1, Supplementary Figure S1). In 2018, each plant in the field was examined for visual symptoms and 100% of the genotypes had at least one symptomatic plant per plot. In 2019, data from only five plants were recorded individually for disease incidence. One hundred percent of the plants visually screened for each genotype had leaf crumple incidence; however, disease severity among genotypes varied considerably (Figure 1, Supplementary Figure S1). None of the genotypes were symptomless or immune in both years tested.

In 2018, of the 84 genotypes, 19 genotypes showed a high level of resistance to leaf crumple, 25 genotypes were moderately resistant, 11 genotypes were susceptible, and 29 genotypes were found to be highly susceptible (Table 1).

In general, disease severities were higher in most of the genotypes in 2019 compared to 2018. Many genotypes that were resistant in 2018 were susceptible in 2019. At 30 DAS, disease severity was higher in most genotypes compared to 45 DAS. Sixteen snap bean genotypes were classified as highly resistant in 2018 and showed higher disease severities in 2019. In 2019, twenty-four snap bean genotypes were moderately resistant, 24 were susceptible and 31 genotypes were highly susceptible (Table 1).

The two Lima bean genotypes (*P. lunatus*), Jackson Wonder and Fordhook had low disease severity in 2018 and 2019 (Table 1). Eight *Phaseolus* genotypes were moderately resistant in 2018 and 2019 with disease severities ranging from 21% to 50% for Barron, Carson, Cedric Larson, Fordhook, Furano, Hastings white cornfield, Hmx 175724 and Wyatt.

### 3.2. Confirmation of Begomoviruses (CuLCrV and/or SiGMFV) Infection in Phaseolus spp.

In 2018 and 2019, both the viruses were detected in the field and were prevalent. In 2018 out of the 40 genotypes tested, at least one virus was detected in 13 genotypes (32.5%) and both viruses were detected in 27 genotypes (67.5%). Among the genotypes that were infected with either of these viruses, seven genotypes had CuLCrV whereas six genotypes had SiGMFV. In 2019, out of the 40 genotypes tested, at least one virus was detected in 21 genotypes (52.5%), while both viruses were detected in 38 genotypes (95%). Among the genotypes that were infected with either of these viruses, 11 genotypes had CuLCrV (27.5%), whereas 10 genotypes had SiGMFV (25%).

### 3.3. Accumulation of CuLCrV and SiGMFV and Leaf Crumple Severity in P. vulgaris Genotypes (Susceptible vs. Resistant; Identified in Field Screen) when Exposed to Viruliferous Whiteflies (Mixed Infected with CuLCrV and SIGMFV) under Greenhouse Conditions

**Figure 2:**
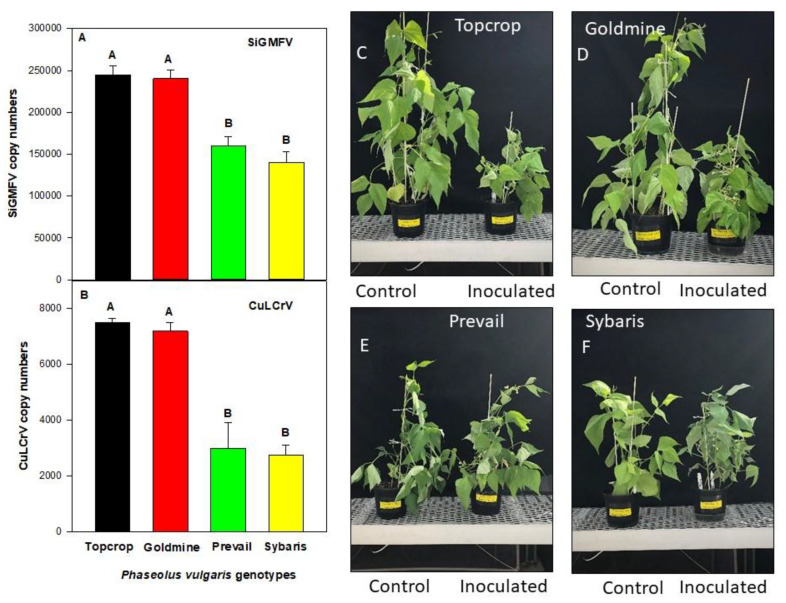
Accumulation of CuLCrV and SiGMFV and leaf crumple severity in *P. vulgaris* genotypes in greenhouse (susceptible vs. resistant; identified in field screen). Data points represent the mean copy numbers of SiGMFV (**A**) and CuLCrV (**B**) in two susceptible (Top crop and Gold mine) and two resistant genotypes (Prevail and Sybaris) in two independent experiments. Bars indicate standard error of the mean. Means with similar letter are not significantly different according to least significant difference (LSD) at *p* = 0.05 level. Response of two susceptible [Top crop (**C**) and Gold mine (**D**)] and two resistant [Prevail (**E**) and Sybaris (**F**)] genotypes to mixed infection (CuLCrV and SiGMFV).

When inoculated plants were sampled in greenhouse at 30 DPI, significantly higher copy numbers of SiGMFV accumulated in the Top crop and Gold mine compared to Sybaris and Prevail (Figure 2A). Additionally, during the same sampling period, significantly higher copy numbers of CuLCrV were accumulated in the Top crop and Gold mine, compared to Sybaris and Prevail (Figure 2B). The viral pathogens were not detected in the genotypes that were inoculated with non-viruliferous whiteflies.

The effect of genotypes on disease severity upon exposure to viruliferous whiteflies (CuLCrV and SIGMFV) was significant (*p* = 0.001). At 30 DPI, disease severity ratings for Top crop (85 ± 7.1%) and Gold mine (85 ± 4.2%) were significantly higher than the ratings for the genotypes Prevail (38.8 ± 8.5%) and Sybaris (35.5 ± 9.8%). None of the genotypes that were exposed to non-viruliferous whiteflies displayed any visible symptoms. A representation of the disease symptoms observed for these four genotypes when exposed to viruliferous and non- viruliferous whiteflies under greenhouse conditions is given in Figure 2C–F.

### 3.4. Whitefly Count

**Figure 3:**
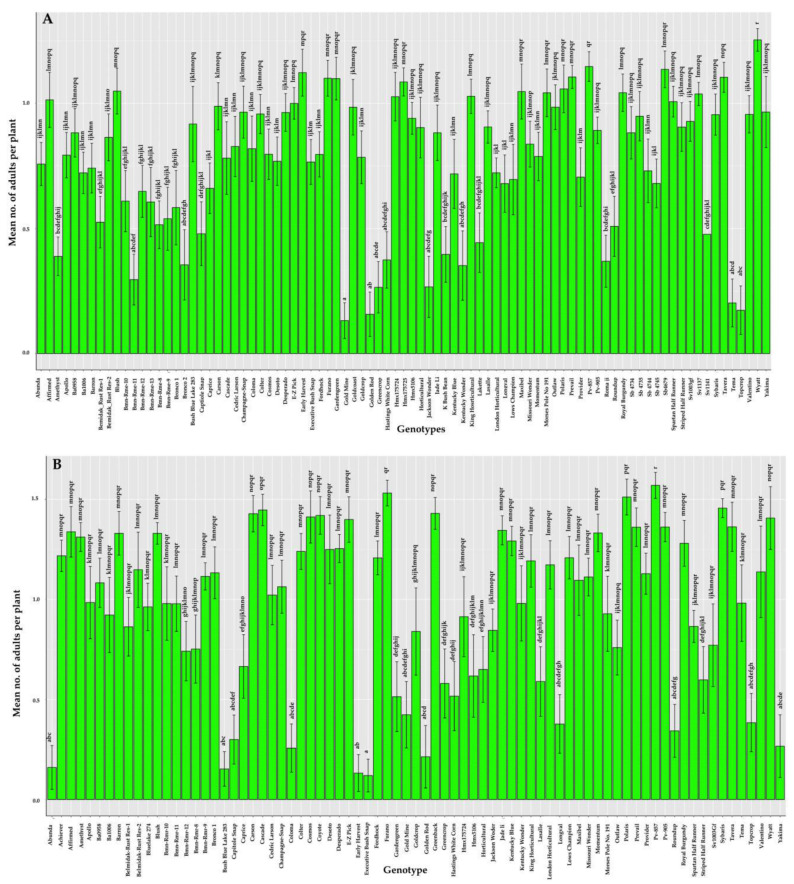
Mean number of whitefly adults counted on different snap bean genotypes in 2018 (**A**) and 2019 (**B**). Bars with standard errors represent the average number of whiteflies present on each plant of the genotype. Whiteflies adults were enumerated on the three top leaves of the plant. Y-axis is shown in a logarithmic scale. Bars with different letters are significantly different from one another (*p* < 0.05).

In 2018, whitefly counts differ significantly between *Phaseolus* spp. genotypes (*F*_(86,1164)_ = 7.12, *p* < 0.001). The genotypes Bmn-Rmr-11, Bronco 2, Gold mine, Golden rod, Jackson wonder, Tema, and Top crop had a significantly lower number of mean adult whitefly counts compared to other genotypes with the lowest count recorded for the Gold mine (Figure 3A). Similarly, in 2019, whitefly counts differed significantly among the genotypes of *Phaseolus* spp. (*F_(_*_76,1078)_ = 9.13, *p* < 0.001). The genotypes Abunda, Bush blue lake 283, Capitole snap, Coloma, Early harvest, Executive bush bean, Golden rod, Longval, Roundup, Top crop and Yakima had significantly lower number of mean adult whitefly counts compared to other genotypes with the lowest count recorded for the Executive bush bean (Figure 3B). The genotypes, Top crop and Golden rod, had consistently lower mean counts of adult whiteflies for two consecutive years.

### 3.5. Data filtering, Mapping and Variants Identification

A total of over six billion raw-read data were generated. Per sample, the raw data generated ranged from a minimum of 29.8 million to a maximum of 73.1 million paired-end reads (Supplementary Table S1, Supplementary Figure S2). The raw read data were quality filtered. Overall, more than 97% data (903.6 Gb) were retained (Table 2, Supplementary Figure S3). The filtered read data were mapped on to the reference genome of *P. vulgaris*. A total of over five billion reads were mapped on the reference genome, amounting to 88.6% mapping rate. Seventy-one *Phaseolus* genotypes showed that more than 75% of filtered reads were successfully mapped (75%–97%); however, 11 genotypes showed less than 75% mapping (Supplementary Figure S3). Details of total number of filtered reads mapped for each sample is listed in the Supplementary Table S2. The average depth (X) of mapped reads at each site ranged from 11.8 to 22.3, calculated based on the total number of bases in the mapped reads divided by size of the assembled genome. Percentage of genome coverage with more than one read mapped (at least 1×) ranged from 46.5% to 96.9%. Percentage of genome coverage with 4× ranged from 39.5% to 94.98%. Overall, Fordhook (55.8%) and Jackson wonder (60. 8%) displayed low mapping because of low genome coverage of 39.5% and 39.19%, respectively (Supplementary Table S2). A total of 21,042,255 raw SNPs and 4,156,878 raw InDels were identified on eleven chromosomes and 467 scaffolds of *P. vulgaris*. Out of these variants initially identified, 20,117,468 biallelic SNPs and 3,732,869 biallelic InDels were retained further. After removing the non-variant sites, 11,572,528 SNPs and 2,465,936 SNPs were retained. Further, applying missing variant site, minor allele frequency (0.10) filters and excluding the variants present on scaffolds, a total of 645,729 SNPs and 68,713 InDels were identified on eleven chromosomes of *P. vulgaris*. 

### 3.6. Analysis and Annotation of SNPs and InDels

**Figure 4:**
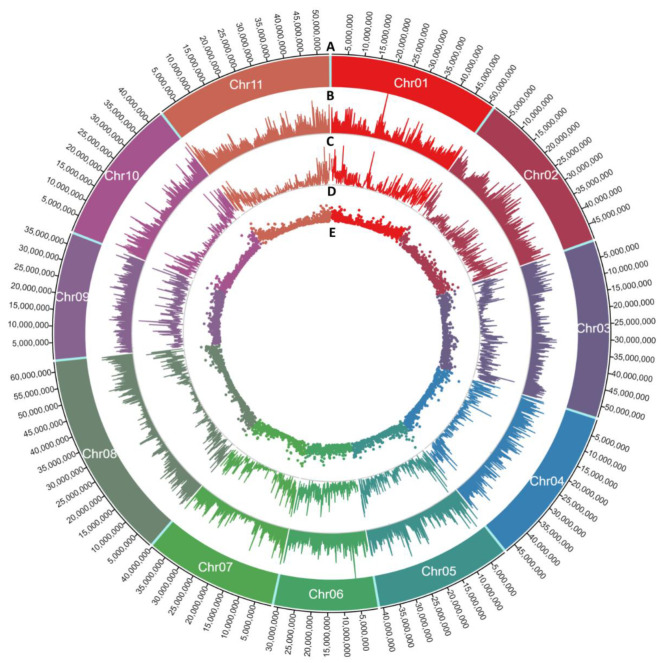
Circos plot to show the density (no. of variants/kb) of SNPs, insertions and deletions in bins of 100 Kb on 11 chromosomes of *Phaseolus vulgaris*. The outermost track (**A**) denotes the physical distance on each of the eleven chromosomes at 5 Mb break-point. Track (**B**) denotes the chromosome numbers. Track (**C**) shows the area plot of SNP density. Track (**D**) represent the line plot of insertion density and Track E represent the scatter plot of deletion density.

**Figure 5:**
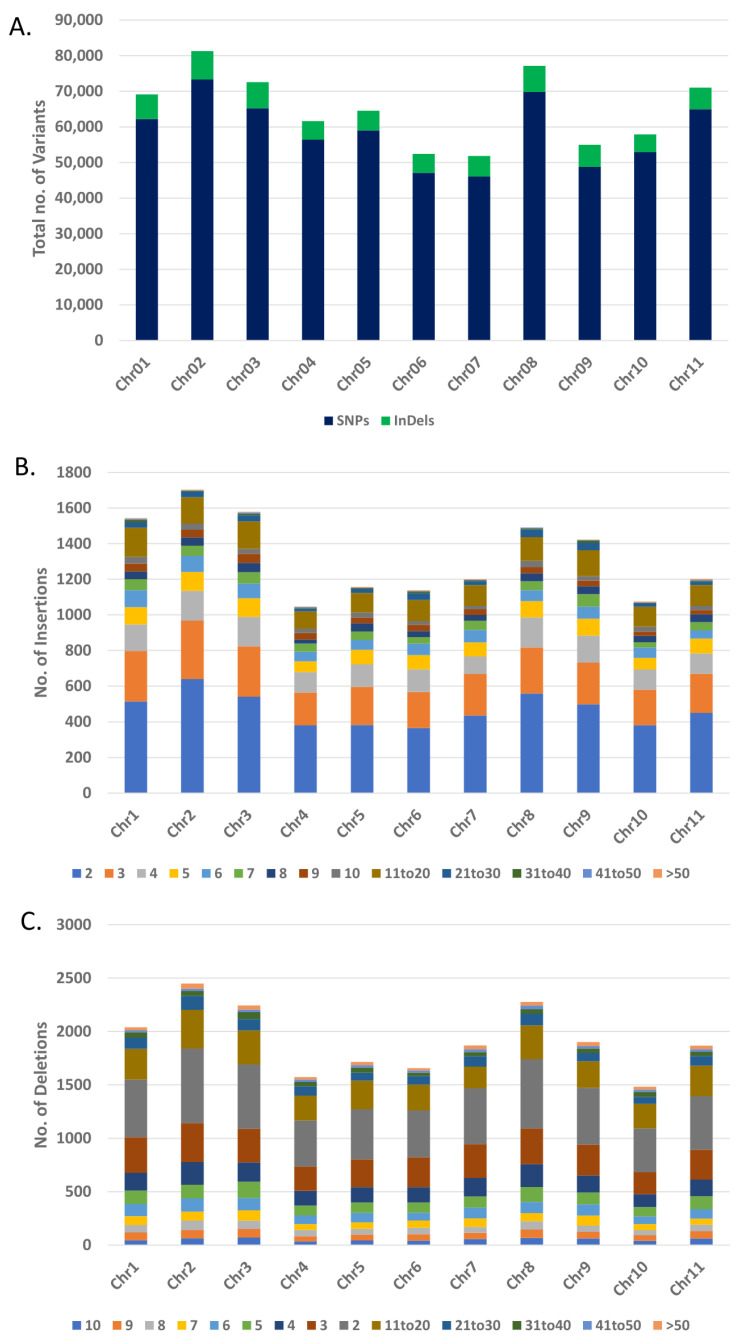
Genome-wide distribution of SNPs and InDels. Chromosome-wise distribution of SNPs and InDels (**A**). Chromosome-wise distribution of indels ranging from 2 bp to >50 bp (**B**). Chromosome-wise distribution of indels ranging from 2 bp to >50 bp (**C**).

SNP and Indel densities varied among the chromosomes. The maximum SNP density of 6.39 SNPs/kb was identified on chromosome 1 (19.2 Mb to 19.3 Mb bin), followed by chromosomes 7 (5.7 Mb to 5.8 Mb bin) and 5 (2 Mb to 2.1 Mb bin) with SNP densities of 5.91/kb and 5.28/kb, respectively (Supplementary Tables S3–S5). Maximum insertion and deletion densities of 0.37/kb and 0.54/kb were identified in the same region of chromosome 5 (3 Mb to 3.1 Mb bin). Overall, five 100 kb bins on chromosomes 1, 2, 5, 6 and 11 were found with density >500 SNPs/100 kb, seven such bins with insertions >30/100 kb were identified on chromosomes 2, 3, 5 (two bins), 9 (two bins) and 10. Similarly, six bins with deletions >40/100 kb on chromosomes 2, 4, 5 (two regions), 7 and 11 were identified (Figure 4, Supplementary Tables S3–S5). A maximum of 73,326 and a minimum of 46,028 SNPs were identified on chromosomes 02 and 07, respectively (Table 3, Figure 5A). Length of a chromosome and number of variants are generally directly correlated i.e., the longest chromosome is expected to possess the greatest number of SNPs and InDels. However, in the current study, we identified the maximum number of SNPs (73,326) on chromosome 02 (the fifth longest chromosome with length 49.67 Mb). On the contrary, Chromosome 08 (the largest with length 63.05 Mb) contained second largest number of SNPs (69,823). Similarly, the greatest number of insertions (3522) and deletions (4429) were found on chromosome 02. The minimum number of Indels were identified on chromosome 10 (Table 3, Figure 5B,C). Length of insertions ranged from 1 to 181 bp and that of deletions ranged from −1 to −109 bp. The number of deletions (38,550) was higher than the number of insertions (30,165) with a maximum frequency of 1 bp insertions and deletions (Table 3, Supplementary Tables S6 and S7). Investigation on the nucleotide substitution type of SNPs indicated higher frequency of transitions (C/T and G/A; Ts = 407,325) than transversions (C/A, G/T, C/G and T/A; Tv = 238,404) and the ratio of Ts/Tv was 1.71 (Table 4).

**Figure 6:**
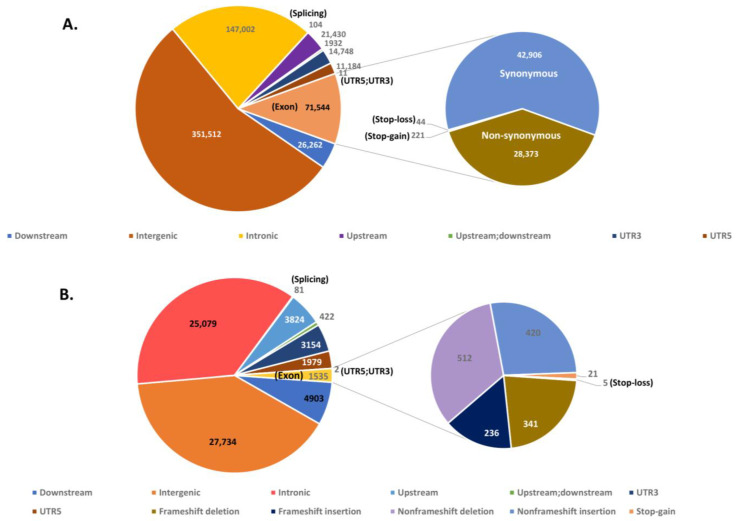
Annotation of single nucleotide polymorphisms (SNPs) and InDels. Distribution of SNPs (A) and InDels (B) in different regions of genome. SNPs in exonic regions are classified into synonymous, non-synonymous, stop-loss and stop-gain categories (**A**). Indels in exonic regions are classified into frameshift deletion, frameshift insertion, nonframeshift deletion, and stop-gain and stop-loss categories (**B**).

Only 71,544 (11%) SNPs were identified in the exonic regions of chromosomes (Figure 6A). The exonic SNPs were further annotated into nonsynonymous (28,373; 39.65%), synonymous (42,906; 59.97%), stop gain (221; 0.003%) and stop loss (44; 0.0006%) SNPs (Figure 6B). Out of 68,715 InDels, only 1535 (2.2%) InDels were identified within the exons on chromosomes. The maximum number of exonic InDels was annotated as non-frameshift deletions (512), followed by non-frameshift insertion (420), frameshift deletion (341), frameshift insertion (236), stop gain (21) and the least, stop loss (5) (Figure 6B). Overall, SNPs and InDels showed similar distribution patterns in the genome. These variants were found in intergenic, intronic, splicing, UTR downstream and upstream region of genes (Figure 6A,B).

## 4. Discussion

A total of 88 different *Phaseolus* genotypes were evaluated for natural resistance to CuLCrV and SiGMFV, with 84 evaluated in 2018 and 80 evaluated in 2019. There were 76 genotypes common in both the years. Further, the 82 genotypes were sequenced, and the SNP and InDel variants were identified. Overall, the aim was to identify both phenotypic variability (symptom severity to CuLCrV and SiGMFV) and diversity within the genomes of these genotypes. All genotypes displayed begomovirus-associated symptoms in the field, suggesting that none of the genotypes were immune. The disease severity ranged from 5 to 100%, indicating a considerable difference in disease resistance among the genotypes. However, we observed some inconsistencies for the phenotypic response of genotypes in 2018 and 2019. For example, the genotypes; Affirmed, Blush, Royal burgundy, Prevail and Tema showed high-to-moderate level of resistance against the leaf crumple disease in 2018 (severity: 5–23%). However, in 2019 the symptom severity for these genotypes ranged from 46–55%. This could be due to the comparatively higher level of infestation with whiteflies in 2019 vs. 2018, resulting in presumably higher inoculation events with one and/or both begomoviruses. Moreover, the percentage of genotypes that were mixed and infected with both begomoviruses were higher in 2019 (95%) vs. 2018 (67.5%), and as per the previous observations, these plants can display severe symptoms compared with when they are infected with either of the viral pathogens [17]. It is possible that more genotypes were mixed infected in 2019 than in 2018 resulting in severe symptoms as observed for the same genotypes earlier. Interestingly, the two Lima bean genotypes, Fordhook and Jackson wonder were highly resistant in both years. Based on the greenhouse evaluation with two genotypes-each (susceptible vs. resistant) exposed to viruliferous whiteflies infected with CuLCrV and SiGMFV, we observed that the susceptible genotypes accumulated higher copy numbers of both viruses and displayed severe crumple severity compared with the resistant genotypes. Although the experiments were done under no-choice scenario, the results indicate that the genotypes might be resistant to the virus complex itself than against the whiteflies. Further detailed greenhouse studies with other genotypes from each phenotypic class (highly resistant vs. highly susceptible vs. moderately resistant) should be conducted to characterize the mechanism of resistance.

Adult whitefly counts differed among the genotypes during both the years, indicating a potential difference in preference to these genotypes or host-related factors that repel whiteflies, which needs to be investigated further. It is also possible that these responses could potentially be due to antibiosis and/or antixenosis resistance to *B. tabaci*, which needs to be evaluated. Carefully planned extensive preference and biology experiments are required to fully comprehend the level of resistance of snap bean genotypes to *B. tabaci*.

The genotypes Top crop and Golden rod had consistently lower mean counts of adult whiteflies for two consecutive years. Interestingly, despite the lower number of whiteflies on these genotypes, these genotypes displayed highly susceptible reactions with disease severity more than 80%. These observations could be due to migration of whiteflies from highly susceptible genotypes with severe symptoms and less green foliage to heathy appearing genotypes for feeding. However, it is unclear if such observations are only due to host-preference or any other host or insect related factors. Further investigation under controlled greenhouse conditions is required to support this proposition.

Next generation sequencing (NGS) technology, particularly the WGS with downstream computational analyses have provided a quick and accurate method to discover genome-wide variations and to identify marker-trait associations, as exemplified in several other studies [18,19,20,21,22,23]. Earlier studies deployed genotyping by sequencing (GBS), which resulted in reduced representation of genome and captured less genomic variants [22] or used much less frequently present simple sequence repeats (SSRs) [24]. We therefore generated WGS data of 82 *Phaseolus* genotypes and aligned it on the *P. vulgaris* reference genome [1]. A wide variation in the total number of sequenced reads was observed (59.6 million to 146.1 million), with a mapping rate ranging from 55.19% to 97.19%. The low mapping rate of genotypes Fordhook and Jackson wonder is due to the fact that these two genotypes belong to *P. lunatus*; however, these were mapped on to the *P. vulgaris* reference genome. The reason for low mapping could lie in the breeding history of these cultivars, which might have resulted in allelic admixture events in these nine *P. vulgaris* genotypes. With an average density of 125 SNPs, five insertions and seven deletions/100 kb variants were differentially distributed throughout the genome. There were several 100 kb bins on each of the 11 chromosomes that did not contain such variants. Despite having uniform genome coverage of mapped reads, several empty bins were identified because of the stringent variant calling parameters used, as indicated in the methods section. Such significant differential distribution of DNA polymorphisms has also been reported in Arabidopsis and rice [25,26,27].

Varshney et al. [28] reported SNP and InDel densities (per 100 kb) of 63.3 SNPs and 38 InDels in cultivated chickpea, and 103.4 SNPs and 67.4 InDels in wild chickpea using 412 cultivated and seven wild chickpea genotypes. We observed a higher SNP but lower InDel density in our 82 genotypes when compared to a cool-season legume crop (chickpea). The variant density is expected to increase even further if we consider a larger set of genotypes for genotyping. This clearly indicates that *Phaseolus* has more genetic diversity than its cool season counterpart that can be deployed for breeding for disease resistance. The identified SNP density in this study (125/100 kb) is also comparable to a warm-season legume, soybean (~100 SNP/100 Kb) [29]. In our study, the ratio of non-synonymous to synonymous SNPs was found to be 0.66, which is less than the ratio observed in pigeon pea (*Cajanus cajan*; 1.18) [30], soybean (*Glycine max*.; 1.36) [29], rice (*Oryza sativa*; 1.18) [31], sorghum (*Sorghum bicolor*; 1.0) [32] and chickpea (*Cicer arietinum*; 1.20) [28]. The lesser ratio in our study indicates that synonymous substitutions in the studied *Phaseolus* genotypes are tolerated, but the non-synonymous substitutions are removed by purifying the selection. It suggests that functionally constrained regions of genes evolve at a slower rate than regions that are not functionally constrained.

The Ts/Tv ratio is often used as a quality indicator of variation data produced from NGS experiments. A higher ratio is an indicator of good quality SNPs, as sequencing errors and false positive variants have a ratio closer to one [33].We found the SNP transitions (A/G and C/T) are the most common substitution in the genome, which is consistent with other crop species like foxtail millet (*Setaria italica*) [34], tea (*Camellia sinensis*) [35], soybean [36], and rice [27]. We observed a Ts/Tv ratio of 1.71 is, however, less than the ratios reported in crops like rice [27], maize (*Zea mays*) [37] and tea [35]. The higher Ts/Tv could be because of more synonymous mutations resulting from from transitions other than transversions, which brings out the change in protein structure and function.

Overall, we identified 20 genotypes (18 snap bean and 2 Lima bean) that consistently displayed high-to-moderate levels of resistance to begomoviruses under field conditions. Greenhouse evaluation also indicates that the genotypic resistance could potentially be against the virus complex rather than against the whiteflies. Further characterization and confirmation of resistance response with a larger set of genotypes should be conducted under controlled greenhouse conditions with standard parameters (exposure to standard or equal number of viruliferous whiteflies). Additionally, it is important to evaluate if the observed responses under field conditions are not due to antibiosis and/or antixenosis resistance to *B. tabaci*. Hence, controlled studies are required to evaluate these factors. Nevertheless, this is the first report of field evaluation of commercially available *Phaseolus* spp. (snap bean and Lima bean) cultivars against natural infection of prevalent begomovirus complex (CuLCrV and SiGMFV) in the southeastern US particularly in Georgia. Further, we identified number of genomic variants in these commercial cultivars that were not reported earlier. These genomic variants will serve as a genetic basis for identifying resistance against these begomovirus complex in *Phaseolus* spp. GWAS studies are underway to identify resistance genes that confer field resistance.

## 5. Conclusions

Our study reports the occurrence of CuLCrV and/or SiGMFV-induced symptoms in *Phaseolus* genotypes, including 80 snap beans and two Lima bean genotypes. Based on our phenotyping experiments in field and genomics assisted studies, we conclude that the tested genotypes depict significant variations in susceptibility against one and/or both viruses. Greenhouse evaluation also indicates that the genotypic resistance could potentially be against the virus complex rather than against the whiteflies. Future comprehensive studies will be carried out with larger sets of *Phaseolus* germplasms, which will aid in associating genetic diversity with diverse disease response against both the begomoviruses.

## Figures and Tables

**Table 1:** Response of *Phaseolus* genotypes to leaf crumple disease in the field during 2018 and 2019 cropping seasons.

Serial number	Genotype	Source	Disease Severity (%)				2019 Mean (%) ± SE	HR/MR/S/HS
			2018 30 DAS	HR ^a^/MR ^b^/S ^c^/HS ^d^	2019 30 DAS ^e^	2019 45 DAS		
1	Abunda *	GRIN	77 ^f^	HS	100	100	100 ± 0	HS
2	Affirmed *	Seminis	17	HR	53	57	55 ± 2	S
3	Amethyst *	Johhny’s seed	20	HR	33	33	33 ± 0	MR
4	Apollo *	GRIN	33	MR	60	60	60 ± 0	S
5	BA0958 *	Jenna	53	S	70	74	72 ± 2	HS
6	BA1006 *	Jenna	77	HS	65	65	65 ± 0	S
7	Barron *	Harris Moran	30	MR	73	27	50 ± 23	MR
8	Belmidak-Rust Resistant-1 *	GRIN	73	HS	60	60	60 ± 0	S
9	Belmidak-Rust Resistant-2 *	GRIN	67	HS	40	37	38.5 ± 1.5	MR
10	BLUSH *	GRIN	17	HR	47	50	48.5 ± 1.5	MR
11	BMN- RMR- 13 *	GRIN	52	S	NS ^g^	NS	_	_
12	BMN-RMR-10 *	GRIN	53	S	63	67	65 ± 2	S
13	BMN-RMR-11 *	GRIN	73	HS	70	77	73.5 ± 3.5	HS
14	BMN-RMR-12 *	GRIN	80	HS	83	86	84.5 ± 1.5	HS
15	BMN-RMR-8 *	GRIN	50	MR	72	80	76 ± 4	HS
16	BMN-RMR-9 *	GRIN	60	S	70	70	70 ± 0	HS
17	Bronco 1 *	Seminis	80	HS	53	57	55 ± 2	S
18	Bronco 2 *	Seminis	90	HS	NS	NS	_	_
19	Bush Blue Lake 283 *	Asgrow Seed Co	80	HS	100	100	100 ± 0	HS
20	Capitole Snap *	GRIN	80	HS	70	73	71.5 ± 1.5	HS
21	Caprice *	Harris Moran	87	HS	100	100	100 ± 0	HS
22	Carson *	Syngenta	22	MR	43	47	45 ± 2	MR
23	Cascade *	GRIN	42	MR	57	59	58 ± 1	S
24	Cedric Larson *	GRIN	27	MR	37	43	40 ± 3	MR
25	Champagne *	GRIN	55	S	47	59	53 ± 6	S
26	Coloma *	GRIN	97	HS	93	100	96.5 ± 3.5	HS
27	Colter *	Harris Moran	27	MR	57	60	58.5 ± 1.5	S
28	Cosmos *	Johnny’s seed	60	S	53	63	58 ± 5	S
29	Desoto *	Harris Moran	20	HR	40	50	45 ± 5	MR
30	Desperado *	Burpee	20	HR	47	47	47 ± 0	MR
31	Early Harvest *	GRIN	80	HS	60	100	80 ± 20	HS
32	Executive Bush Snap *	GRIN	87	HS	100	100	100 ± 0	HS
33	E-Z pick *	Johhny’s seed	82	HS	97	99	98 ± 1	HS
34	Fordhook *	Seedway	23	MR	25	18	21.5 ± 3.5	MR
35	Furano *	Syngenta	22	MR	32	40	36 ± 4	MR
36	Gardengreen *	GRIN	33	MR	67	83	75 ± 8	HS
37	Gold Mine *	Seminis	87	HS	100	100	100 ± 0	HS
38	Goldcoast *	GRIN	67	HS	100	100	100 ± 0	HS
39	Goldcrop *	GRIN	17	HR	47	47	47 ± 0	MR
40	Greencrop *	Seedway	80	HS	88	67	77.5 ± 10.5	HS
41	Hastings White Cornfield *	GRIN	35	MR	45	45	45 ± 0	MR
42	Hmx175724 *	Harris Moran	27	MR	50	47	48.5 ± 1.5	MR
43	Hmx5106 *	Harris Moran	12	HR	47	46	46.5 ± 0.5	MR
44	Horticultural *	Seedway	93	HS	81	90	85.5 ± 4.5	HS
45	Jackson Wonder *	GRIN	5	HR	23	12	17.5 ± 5.5	HR
46	Jade II *	Harris Moran	40	MR	57	57	57 ± 0	S
47	Kentucky Blue *	Sieger	50	MR	70	72	71 ± 1	HS
48	Kentucky Wonder *	Seedway	35	MR	67	68	67.5 ± 0.5	HS
49	King Horticultural *	GRIN	40	MR	60	65	62.5 ± 2.5	S
50	Lakatte *	GRIN	93	HS	NS	NS	_	_
51	Lasalle *	Harris Moran	80	HS	95	100	97.5 ± 2.5	HS
52	London Horticultural *	GRIN	33	MR	50	57	53.5 ± 3.5	S
53	Longval *	GRIN	87	HS	77	95	86 ± 9	HS
54	Lows Champion *	GRIN	17	HR	67	43	55 ± 12	S
55	Maxibel *	Johhny’s seed	57	S	53	57	55 ± 2	S
56	Missouri Wonder *	GRIN	57	S	60	80	70 ± 10	HS
57	Momentum *	Syngenta	20	HR	37	40	38.5 ± 1.5	MR
58	Morses Pole No 191 *	GRIN	53	S	53	53	53 ± 0	S
59	Outlaw *	Stokes seeds	73	HS	47	63	55 ± 8	S
60	Polaris *	GRIN	40	MR	66	70	68 ± 2	HS
61	Prevail *	Syngenta	13	HR	45	47	46 ± 1	MR
62	Provider *	Seedway	93	HS	95	97	96 ± 1	HS
63	PV-857 *	Seedway	20	HR	35	37	36 ± 1	MR
64	PV-905 *	PopVriend	27	MR	53	53	53 ± 0	S
65	Roma II *	Seedway	93	HS	80	100	90 ± 10	HS
66	Roundup *	GRIN	80	HS	80	87	83.5 ± 3.5	HS
67	Royal Burgundy *	Johhny’s seed	23	MR	60	43	51.5 ± 8.5	S
68	SB4679 *	GRIN	17	HR	NS	NS	_	_
69	SB4734 *	GRIN	20	HR	NS	NS	_	_
70	SB4735 *	GRIN	50	MR	NS	NS	_	_
71	SB4744 *	GRIN	37	MR	NS	NS	_	_
72	Spartan Half Runner *	GRIN	53	S	40	40	40 ± 0	MR
73	Striped Half Runner *	GRIN	33	MR	79	39	59 ± 20	S
74	SV1003GF *	Stokes seed	20	HR	70	40	55 ± 15	S
75	SV1137 *	GRIN	63	S	NS	NS	_	_
76	Sybaris *	Seminis	13	HR	35	37	36 ± 1	MR
77	Tavera *	Johhny seed	53	S	43	53	48 ± 5	MR
78	Tema *	Semins	5	HR	47	50	48.5 ± 1.5	MR
79	Topcrop *	Seedway	100	HS	77	83	80 ± 3	HS
80	Valentino *	Stokes seed	17	HR	66	45	55.5 ± 10.5	S
81	Wyatt *	Harris Moran	37	MR	37	40	38.5 ± 1.5	MR
82	Yakima *	GRIN	20	HR	53	57	55 ± 2	S
83	Achiever	Dave’s garden	NS	_	53	57	55 ± 2	S
84	Bluelake 274	Ferry Morse	NS	_	75	75	75 ± 0	HS
85	Coyote	Syngenta	NS	_	45	47	46 ± 1	MR
86	Golden Rod	Seminis	77	HS	100	100	100 ± 0	HS
87	Greenback	Seedway	NS	_	40	40	40 ± 0	MR
88	K Bush Bean	GRIN	83	HS	97	100	98.5 ± 1.5	HS

Lima beans (*Phaseolus lunatus*); * Genotypes sequenced; ^a^ Highly resistant; ^b^ Moderately resistant; ^c^ Susceptible; ^d^ Highly susceptible; ^e^ DAS: days after sowing; ^f^ Mean disease severity from 15 plants, five each from three replicated plots; ^g^ NS: not evaluated.

**Table 2:** Overview of raw data generated, data retained after quality filtering and reads mapped on the reference genome.

Total Raw Reads	Filtered Clean Reads	Filtered Data (Gb)	Total Reads Mapped	Av Reads Mapped (%)
6,033,783,354	6,026,076,892	903.6	5,204,929,327	88.59

**Table 3:** Distribution of SNPs and InDels on eleven chromosomes of *Phaseolus vulgaris*.

Chromosome No.	Size (Mb)	No. of SNPs	No. of Insertions	No. of Deletions	No. of InDels
Chr01	51.43	62,199	3156	3766	6922
Chr02	49.67	73,326	3522	4429	7951
Chr03	53.44	65,222	3241	4094	7335
Chr04	48.05	56,422	2249	2928	5177
Chr05	40.92	59,007	2383	3146	5529
Chr06	31.24	47,079	2339	3014	5353
Chr07	40.04	46,028	2500	3301	5801
Chr08	63.05	69,823	3144	4177	7321
Chr09	38.25	48,746	2830	3414	6244
Chr10	44.30	52,900	2226	2793	5019
Chr11	53.58	64,977	2575	3488	6063
Total	513.97	645,729	30,165	38,550	68,715

**Table 4:** Number of transitions and transversions based on the SNPs identified in 82 lines of *Phaseolus* species based on the reference genome of *Phaseolus vulgaris*.

Substitution Type	Substitution	Count
Transversions (Tv)	C/G	46,390
	G/T	59,644
	A/C	59,095
	A/T	73,275
Transitions (Ts)	A/G	204,568
	C/T	202,757
Ratio	Ts	407,325
	Tv	238,404
	Ts/Tv	1.71

## Data Availability

The whole genome sequencing data generated and analyzed in this study is available in NCBI under BioProject ID PRJNA680977

## Supplementary Materials

The following are available online at https://www.mdpi.com/2075-4450/12/1/49/s1, Figure S1: Response of bean genotypes to leaf crumple disease in the field in 2019. Genotype names from left to right- A: Jackson Wonder (5); B: Fordhook (18); C: Furano (40); D: Spartan half runner (40); E: Cascade and F: Caprice (100). Figures in parenthesis are mean disease severity values at 45 days after sowing. Figure S2: Overview of raw data generated and data retained for after quality filtering of 82 lines of *Phaseolus* species for mapping and downstream analyses. Overall >97% of data was retained after quality filtering of raw data. Figure S3: Read mapping statistics of filtered data of 82 lines of *Phaseolus* species on to the reference genome of *Phaseolus vulgaris*. Fourteen out of the 82 Phaseolus lines showed <75% mapping. Table S1: Summary of raw read data generated and amount of clean data obtained after filtering the raw data. Table S2: Total number of filtered reads obtained the number of reads mapped on to the reference genome with mapping percent, average depth and coverage distribution. Table S3: SNP density (no. of SNPs/Kb) calculated in bins of 100 Kb throughout the genome on all eleven chromosomes. Table S4: Insertion density (no. of SNPs/Kb) calculated in bins of 100 Kb throughout the genome on all eleven chromosomes. Table S5: Deletion density (no. of SNPs/Kb) calculated in bins of 100 Kb throughout the genome on all eleven chromosomes. Table S6: Frequency of length distribution of insertions among the 11 chromosomes. Table S7: Frequency of length distribution of deletions among the 11 chromosomes.
