# Supplementary material for: Field Screen and Genotyping of Phaseolus vulgaris against Two Begomoviruses in Georgia, USA
Source: Insects. 2021 Jan 10;12(1):49. doi: 10.3390/insects12010049 (PMC7827361; doi:10.3390/insects12010049)
Supplement: Supplementary file 1 [file insects-12-00049-s001.zip › insects-993363-supplementary/insects-993363-supple-proof.docx]

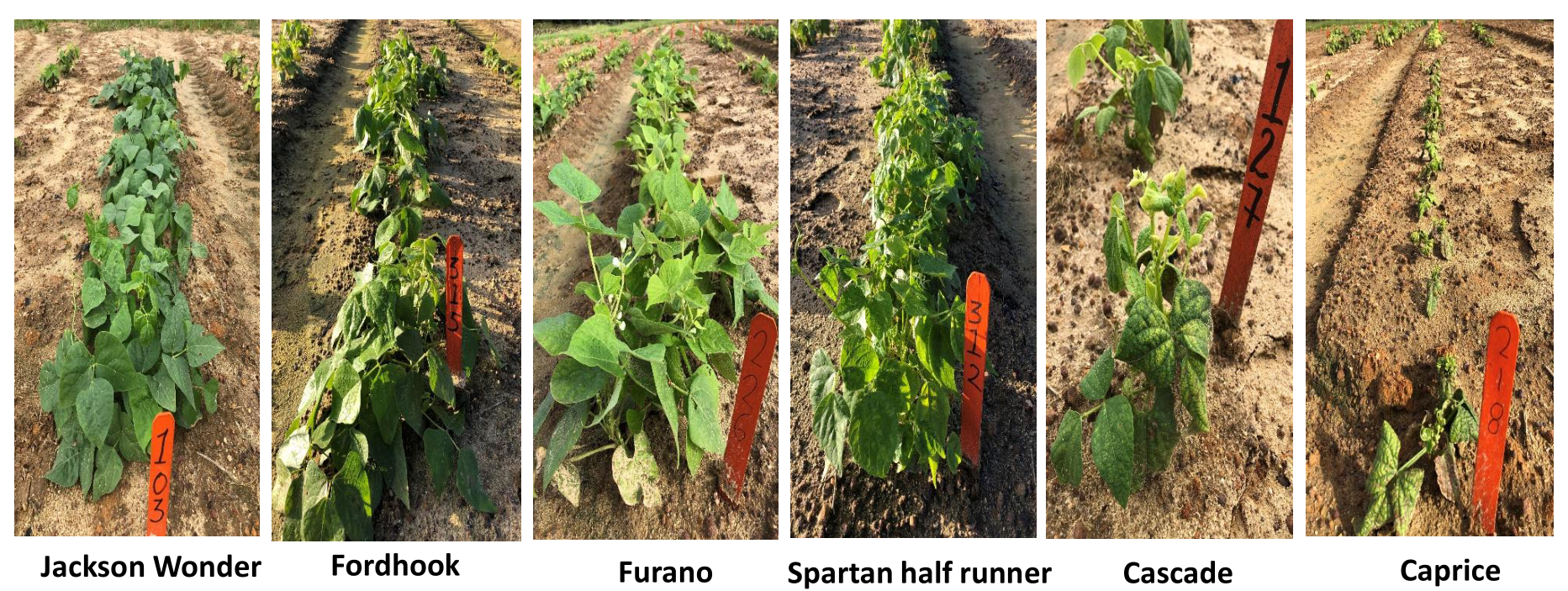


**Figure S1.** Response of bean genotypes to leaf crumple disease in the field in 2019. Genotype names from left to right- A: Jackson Wonder (5); B: Fordhook (18); C: Furano (40); D: Spartan half runner (40); E: Cascade and F: Caprice (100). Figures in parenthesis are mean disease severity values at 45 days after sowing.


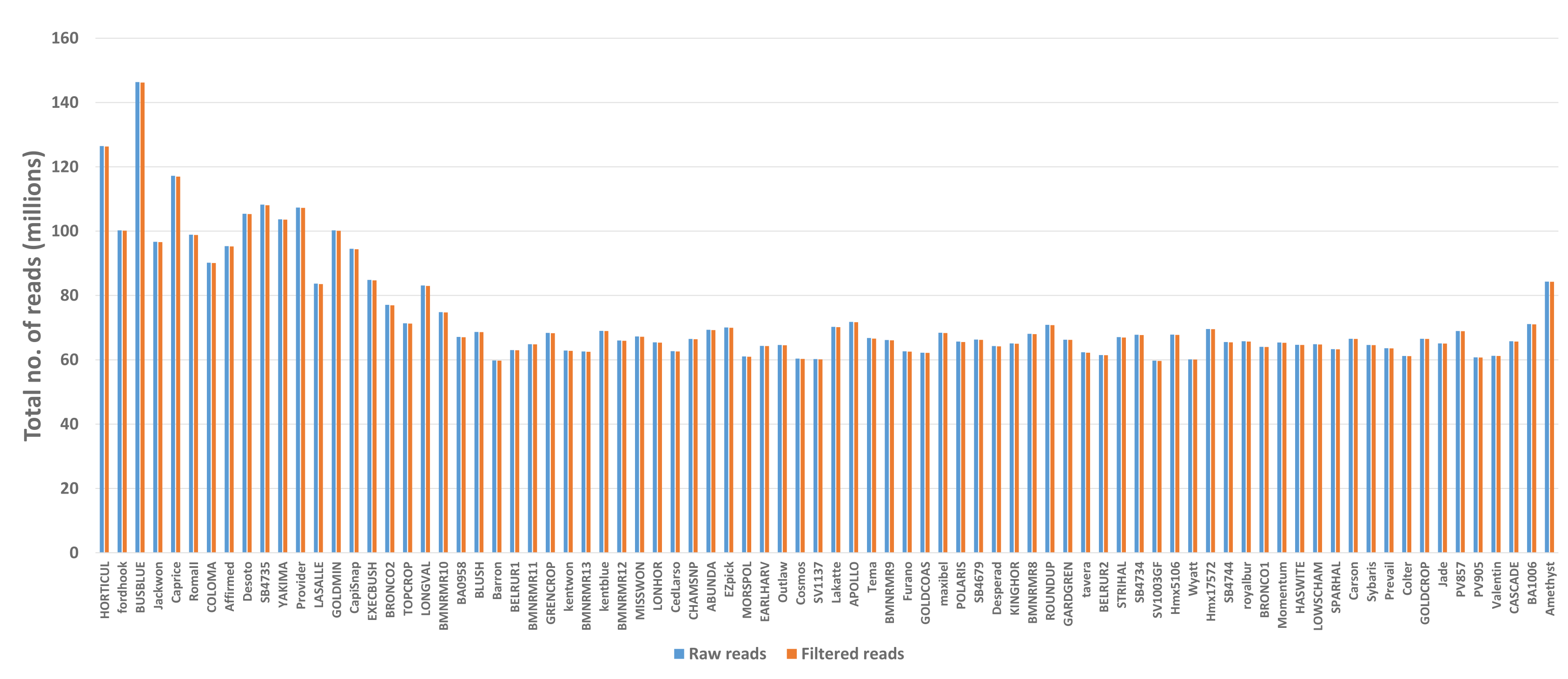


**Figure S2.** Overview of raw data generated and data retained for after quality filtering of 82 lines of *Phaseolus* species for mapping and downstream analyses. Overall >97% of data was retained after quality filtering of raw data.


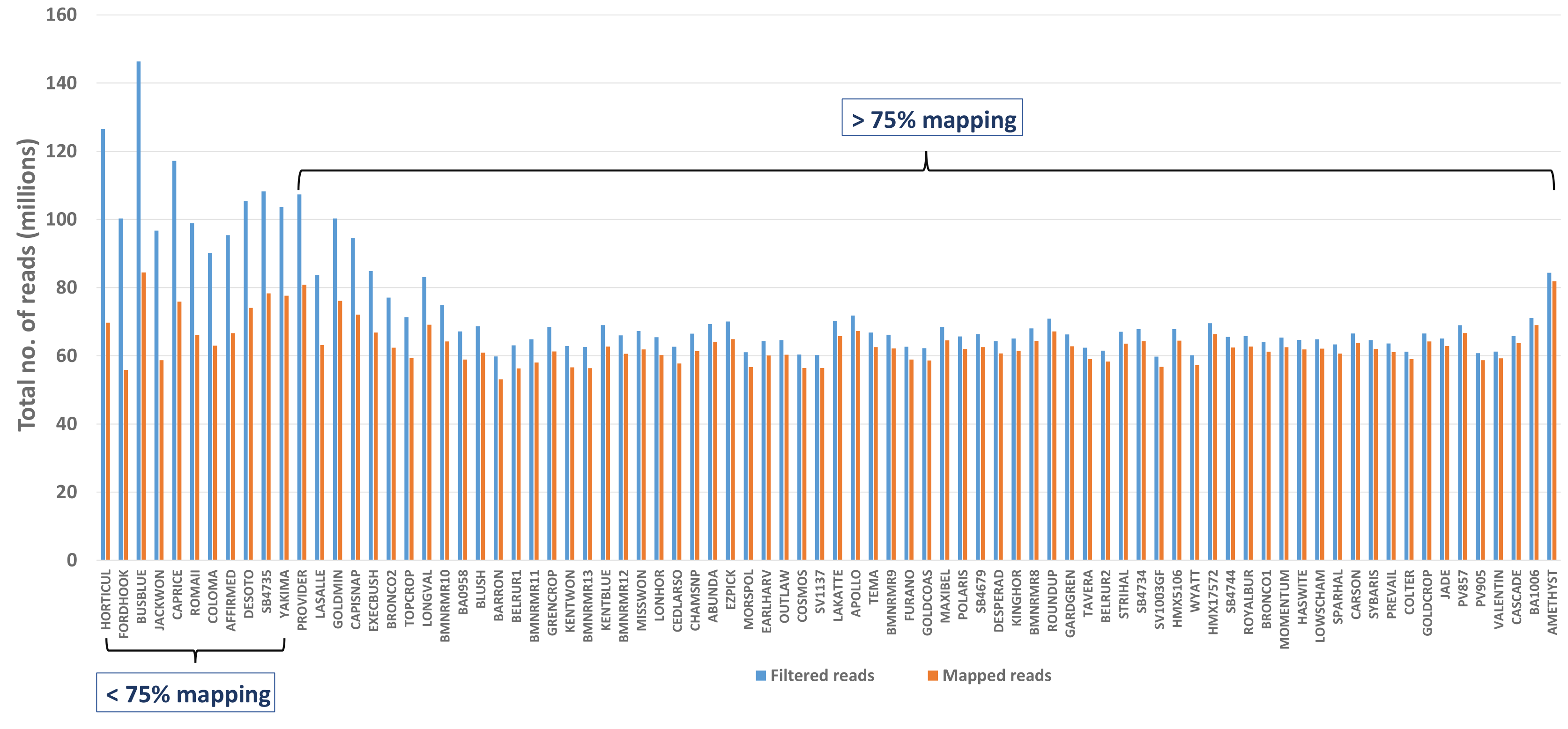


**Figure S3.** Read mapping statistics of filtered data of 82 lines of *Phaseolus* species on to the reference genome of *Phaseolus vulgaris*. Fourteen out of the 82 Phaseolus lines showed <75% mapping.
